# Multifaceted analyses disclose the role of fruit size and skin-russeting in the accumulation pattern of phenolic compounds in apple

**DOI:** 10.1371/journal.pone.0219354

**Published:** 2019-07-15

**Authors:** Nicola Busatto, Daiki Matsumoto, Alice Tadiello, Urska Vrhovsek, Fabrizio Costa

**Affiliations:** 1 Department of Genomics and Biology of Fruit Crops, Research and Innovation Centre, Fondazione Edmund Mach (FEM), San Michele all'Adige, Italy; 2 Faculty of Agriculture, Department of Food, Life, and Environmental Sciences, Yamagata University, Tsuruoka, Japan; 3 Department of Biology, University of Padova, Padova, Italy; 4 Department of Food Quality and Nutrition, Research and Innovation Centre, Fondazione Edmund Mach (FEM), San Michele all'Adige, Italy; ICAR- Indian Agricultural research Institute, INDIA

## Abstract

Fruits are nowadays considered important suppliers of anti-oxidant molecules. Apples are particularly rich in phenolic compounds, non-nutritional phytochemicals that play active roles in controlling severe chronic diseases. In this work, 19 phenolic compounds were investigated in both skin and pulp tissues of seven apple accessions across the *Malus* genus collected at two stages: during fruit development and at harvest. The primary difference in phenolic concentration between wild and domesticated accessions, especially in the pulp, could be explained by the larger growth rate of the domesticated varieties. The proposed dilution effect was also confirmed through the observation of the increased content of procyanidin B2+B4 and phloridzin in russet-skinned apples, known to have higher concentrations of these compounds. The metabolite screening was also accompanied by the expression analysis of 16 polyphenolic genes showing, for nine elements, a higher expression at harvest than during fruit development. Finally, a polyphenolic comparison with red-fleshed apples was also carried out, underlying a larger amount of procyanidins and quercetin-3rhamnoside in the white-fleshed accessions. The results presented and discussed in this work suggest that specific white-fleshed apples, especially with russeted-skin, may play an important role in ameliorating the nutraceutical potential of apple fruit.

## Introduction

Fruits have always been considered relevant components of the daily human diet as natural providers of important nutrient constituents, such as sugars, vitamins, fibers and organic acids[1,2]. These hedonistic properties, together with the external appearance and intrinsic physical features (such as fruit texture), contributed to the formation of the so-called principal quality factors (PQF[3,4]), which are nowadays used as fruit quality descriptors. Out of the four PQFs, the first three are fundamentally related to appearance, flavor and texture. With the recent technological advances in metabolite analysis and biochemistry another PQF was reviewed, the nutritional properties, which is now highly considered by both fruit scientists and consumers[5]. Fruits, nowadays, are in fact appreciated also for their content in important phytochemicals with antioxidant properties[6–13]. These non-nutritional molecules with biological activities, such as polyphenols, carotenoids and vitamins, elevated the fruits to functional commodity essential to promote the human health. While these compounds can affect fruit quality, conferring bitterness and astringency, their main role is assigned to the prevention of important chronic diseases, such as cardio-vascular disorder, diabetes and cancer[14–21]. These bioactive substances are represented by a myriad of compounds, but phenols with antioxidant properties are the most relevant phytochemicals in plant species[11,22–25].

Amongst fruits, apples are particularly rich in phenolic compounds, which are a large group of molecules originated from the aromatic amino acid phenylalanine and tyrosine through the shikimate-derived phenylpropanoid pathway[10,26,27]. Polyphenols are distinguished by more than one phenolic ring[5,13]. In fruit, especially in apple, phenolic compounds can be distinguished into phenolic acids and flavonoids on the basis of their skeleton structure[23,28,29]. Within the category of phenolic acids, one of the most relevant in apple is the chlorogenic acid. Flavonoids, on the other hand, include several distinct compounds, such as flavonols (i.e. quercetin), flavan-3-ols or flavanols (catechin and epicatechin), hydroxycinnamates (coumaric acids, 5’-caffeoyl quinic acid), dihydrochalcone (phloridzin) and anthocyanins[30–34]. There is a growing interest in the protective role exerted by these compounds on the human health, principally due to their redox capacity that enables the quenching of singlet oxygen molecules and the scavenging of free radicals and reactive oxygen species[35,36] (ROS). The investigation of the array of polyphenolic compounds in apple[10,28,34,37,38] was supported by the fact that, upon consumption, polyphenols may promote the human health through the modification of these molecules into other bioactive compounds or directly interacting with the gut microbiota[39,40]. Moreover, it has been shown that the concentration of polyphenolic compounds in human biofluids is not limited by their concentration in fruits[41]. The enhancement of the concentrations of phenolic compounds today is a common valuable target for breeding programs aiming to select ‘fortified’ apple varieties. This would however represent a change in the priority of breeding, focused till now on the release of high quality and attractive type of fruit, but at the same time distinguished by a low concentration of phenolic compounds due to their astringent taste and rapid enzymatic browning[42].

With the aim of facilitating the breeding for polyphenolic compounds through marker assisted selection, several quantitative trait locus (QTL) mapping studies have been carried out. Khan et al.[43] and Chagné et al.[44] identified a number of QTLs potentially involved in the genetic control of these compounds, and identified the *leucoanthocyanidin reductase (LAR1)* gene as one of the major players in controlling flavanol biosynthesis. Khan and colleagues, moreover, underlined a distinct QTL detection with regards to the different metabolite accumulation in peel and flesh, respectively. Similar research was performed to identify QTLs to assist the selection of apple varieties suitable for cider production[45]. Molecular markers associated with novel and important traits for introgression into elite genetic backgrounds are an essential tool for breeders, especially when the donor parent is a low-quality, heritage or wild apple accession. To this end, red-fleshed apples represent the most advanced effort in enhancing the level of anthocyanins in fruit. From a wild red-fleshed accession, molecular markers related to a *MYB* transcription factor have been developed and used to pre-select red-fleshed seedlings[46]. Taking the example of the breeding scheme designed for the selection of red-fleshed apples[47], wild *Malus* species tend to be better parental candidates to increase antioxidant properties in future apple accessions, since wild species are usually richer in phenolic compounds[48–50]. The accumulation of polyphenolic compounds was also assessed together with the expression profile of candidate genes involved in related biochemical pathways in leaf and fruit of apple[51] as well as in fruit of strawberry[52].

In this study, we investigated the accumulation of phenolic compounds comparing skin and pulp tissues in both wild and domesticated accessions at two stages. The quantification of these compounds was compared with the expression pattern of genes representing key steps in the phenolic pathway. The accumulation pattern of phenolic compounds was also analyzed in relation to two pomological features, fruit size and skin russeting, as well as in comparison with red-fleshed apples.

## Materials and methods

### Plant material

The content of phenolic compounds was assessed in seven apple accessions collected at the experimental orchard of the Fondazione Edmund Mach (Trento, Italy): two wild species (*M*. *sieversii* and *M*. *baccata*) and four *M*. x *domestica* accessions, represented by one heritage variety (‘Tyroler Spitzlederer’) and three commercial cultivars (‘Golden Delicious’, ‘Cripps Pink’ and ‘Braeburn’). Of the cultivar ‘Golden Delicious’, two clones were investigated, clone B13 and ‘clone Rugiada’, distinguished by their smooth and totally russeted skin types, respectively. The fruit from each accession (five years old at the time of the analysis) were collected at two specific stages, during fruit development (74 DAFB-days after full bloom for all the accessions employed in this survey) and at the time of the commercial harvest: 144 DAFB for both clones of ‘Golden Delicious’, 159 DAFB for ‘Braeburn’, 187 DAFB for ‘Tyroler Spitzlederer’ and 193 DAFB for ‘Cripps Pink’, established according to the commercial harvest date decided on the basis of the change in fruit firmness, skin and seed color. For the two wild species, fruit were sampled at 110 DAFB for *M*. *sieversii* and at 129 DAFB for *M*. *baccata*, based on skin and seed color and the beginning of fruit drop. At each harvest, 10 apples were collected from each accession, and prior to any molecular and metabolite analysis the fruit weight and diameter were assessed. From each fruit, the skin and skin-free pulp were accurately separated, immediately frozen in liquid nitrogen and stored at -80°C for subsequent biochemical screening and gene expression analysis. In addition, secondary metabolites were assessed at the time of harvest also in three red-fleshed apple accessions, two belonging to wild species (*M*. *pumila* and *M*. *sylvestris*) and one to the *M*. x *domestica* group. The fruit of these accessions, also available in the same collection at the Fondazione Edmund Mach, were harvested following the same parameters applied to the white-fleshed apples.

### Phenolic compound analysis

Both skin and pulp tissues were used to extract and quantify the content of phenolic compounds with the method described by Vrhovsek et al.[53]. Two grams of powdered tissue (obtained grinding the tissue with liquid nitrogen and stored at -80°C until use) were extracted with a solution of water/methanol/chloroform (20:40:40). After separation by centrifuge (1000g at 4°C for 10 min) the aqueous/methanol extract phase was used for a second round of extraction with 2.4 ml of water/methanol (1:2). After centrifugation, the upper phase of two extractions were combined into one to a total volume of 10 ml and filtered with a 0.2 μm PTFE filter. The separation procedure of the phenolic compounds is detailed by Vrhovsek et al.[53] and Busatto et al.[54]. Ultra-high performance liquid chromatography was performed employing a Waters Acquity UPLC system (Milford, MA, USA) coupled with a Water Xevo TQMS (Milford, MA, USA) in ESI ionization mode. Phenolic compounds were separated employing a Waters Acquity HSST3 column 1.8 μm, 100 mm x 2.1mm (Milford, MA, USA) with two solvents: A (water with 0.1% formic acid) and B (acetonitrile with 0.1% formic acid). The separation flow was 0.4 ml/min with a gradient profile of 0 m, 5% B, from 0 to 3 min, linear gradient to 20% B, from 3 to 4.3 min, isocratic 20% B, from 4.3 to 9 min, linear gradient to 45% B, from 9 to 11 min, linear gradient to 100% B, from 11 to 13 min, wash at 100% B, from 13.1 to 15 min, back to initial condition of 5% B. Two μl of both standard solution and samples were injected. Samples were maintained at 6°C during the mass spectrometry detection, carried out with a Waters Xevo TQMS (Milford, MA, USA) equipped with an electrospray ESI source. Capillary voltage was 3.5 KV in positive mode and -2.5 KV in negative mode. Each compound was analyzed with the optimized MRM condition, as detailed by Vrhovsek et al.[53].

### Gene expression analysis by RT-qPCR

From skin and pulp tissues, the total RNA was extracted with the Spectrum Plant total RNA kit (Sigma-Aldrich) and further controlled for quantity and quality with a NanoDrop ND-8000 (ThermoFisher Scientific, MA, USA) and a 2100 Bioanalyzer (Agilent Technologies, CA, USA), respectively. Two μg of extracted RNA were further treated with 2 units of Ambion DNAse and further converted into cDNA with the “SuperScript” VILO cDNA Synthesis kit (ThermoFisher Scientific, MA, USA). The expression of sixteen genes (S1 Table) was determined with a ViiA7 using the FAST SYBR GREEN MASTER MIX (ThermoFisher Scientific, MA, USA). Real Time qPCR reaction was performed with the following thermal profile: 95°C 20 sec, 40 cycles of 95°C 1 sec and 60°C 20 sec and finishing with a final cycle at 95°C 15 sec, 60°C 1 min and 95°C 15 sec, in order to define the reaction melting curve. The gene expression was reported by the mean normalized expression through the use of equation 2 of the “Qgene” software[55].

### Data analysis

The data were analyzed using the software R[56]. The PCA and the Correlation plots were realized using ChemometricsWithR and Corrplot packages, respectively. The heatmaps depicting the gene expression data combined with the polyphenol quantifications were calculated and visualized trough Gene Cluster 3.0 and Java Tree software, respectively[57,58]. Metabolite profiles were processed using the Water MassLynx 4.1 and Target Lynx software.

## Results and discussion

### Fruit size change during development and maturation

**Fig 1:**
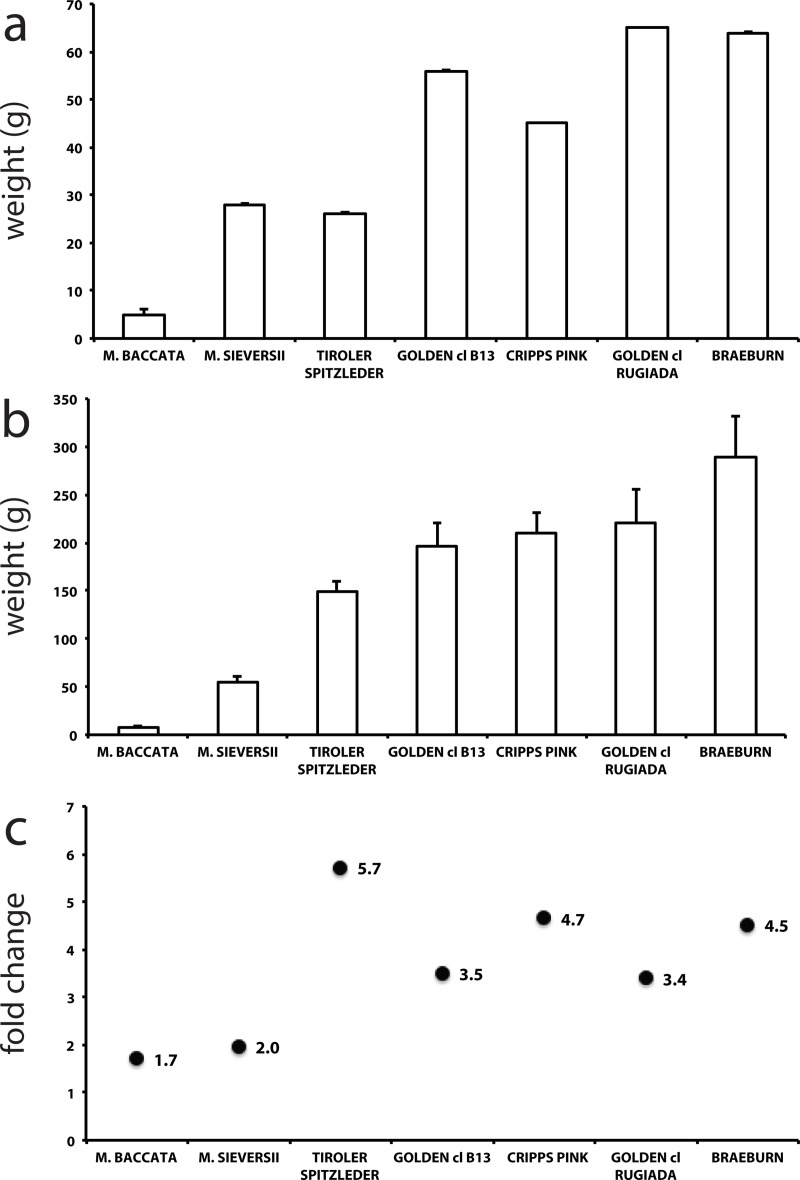
Average fruit weight of the seven apple accessions. The average fruit weight (g) of seven apple accessions measured at fruit development and harvest is depicted in panel a and b, respectively. The fold-change between the two harvesting stages is instead illustrated in panel c. Bars stand for standard errors.

Fruit from the seven accessions employed in this study were sampled at two time points: during fruit development and at the time of commercial harvest. The fruit development date was identified as a single data point, with the fruit of the commercial accessions showing a mean diameter of approximately 3 cm and a similar fruit weight (Fig 1A), ranging from 46 g for ‘Cripps Pink’ to 65 g for ‘Golden Delicious cl. Rugiada’. The different growing rates of the heritage apple variety ‘Tyroler Spitzlederer’ (26 g) and the two wild species were distinctly lower, but ‘Tyroler Spitzlederer’ showed a large growth pattern, with a fruit size closer to the commercial cultivars at harvest (Fig 1B). The higher fruit size of *M*. *sieversii* with regards to *M*. *baccata* find consistency with the hypothesis previously formulated that *M*. *sieversii* is the most probable progenitor of the *M x domestica* species known today[59]. Mostly the same fruit size pattern was observed at the time of commercial harvest (Fig 1B), with a more evident distinction between the wild species (8.2–55.1 g) and the domesticated varieties (148–245 g). The latter showed a 3.4 to 5.7-fold increase in fruit size, whereas it was less than 2-fold for the two wild species (Fig 1C). This may reflect that fruit size was probably the first and most relevant trait improved through the evolution and selection process occurred in apple, as suggested by Doebley et al.[60]. This hypothesis finds also agreement with the study of Duan et al.[61] that through the re-sequencing of 117 diverse accessions of both wild and domesticated species identified genomic regions characterized by a selective sweep for fruit weight.

### Phenolic compound characterization in fruit of apple

**Fig 2:**
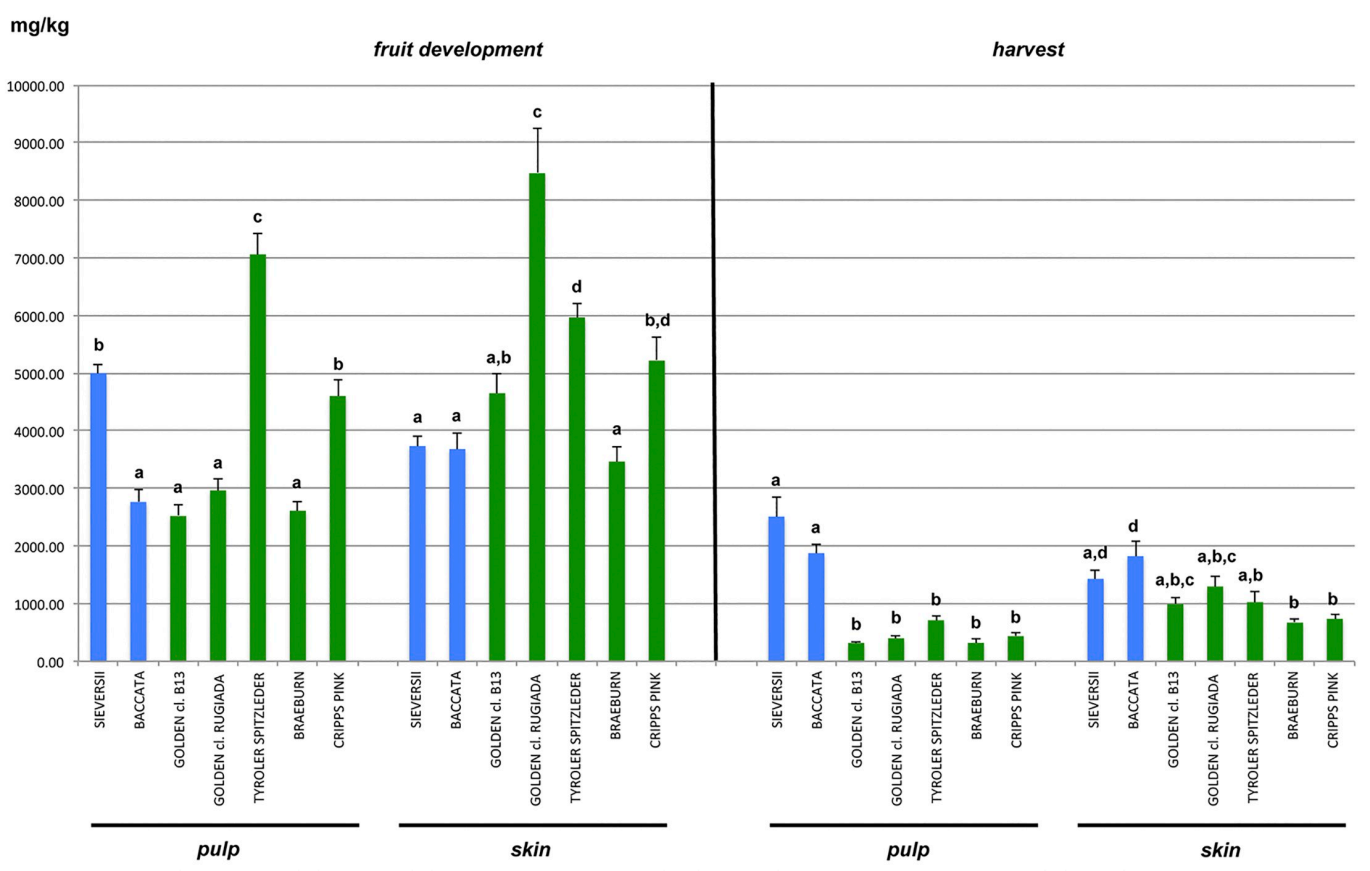
Analysis of phenolic compounds in apple. Total amount of phenolic compounds (mg/kg of fresh weight) in the pulp and skin of the seven apple accessions collected at both fruit development and harvest stage. The types of apple accessions are distinguished by colors, with blue for the wild accessions and green for the domesticated cultivars. Letters indicate difference statistically significant on the base of the Tukey HSD test with an adjusted P*value ≤* 0.01.

The targeted screening of secondary metabolites identified 19 phenolic compounds belonging to seven classes: phenolic acid (vanillin and vanillic acid), hydroxycinnamic acid (neochlorogenic acid, cryptochlorogenic acid and chlorogenic acid), phenylpropanoid (coniferyl alcohol), stilbenes (*trans*-piceid, *cis*-piceid), dihydrochalcones (phloridzin), flavanols or flavan-3-ols (catechin, epicatechin, procyanidin B1, procyanidin B2+B4) and flavonols (quercetin-3-rhamoside, quercetin-3-rutenoside, kampferol-3-rutinoside, isorhammetin-3-glc, rutin and arbutin). From a general overview of the phenolic compound accumulation pattern, two general trends were observed. Phenolic compounds, in general, are more abundant in apples collected during the fruit development stage and more concentrated in skin tissue rather than pulp (S2 Table and Fig 2), in agreement with previous studies[5,42,51]. The fold-change of the total polyphenolic compounds between fruit development and harvest ranged from 1.4 to 10.5 for the pulp and from 2 to 7.1 for the skin tissues. Although fruit skin is generally richer in polyphenols than pulp, with a fold change ranging from 1.1 to 2.8 and from 1.4 to 3.1 for the fruit development and harvest stages, respectively, a few exceptions were observed. At the stage of fruit development, the total polyphenolic concentration in the pulp of *M*. *sieversii* and ‘Tyroler Spitzlederer’ (4997.51 and 7060.92 mg/kg of fresh weight (FW), respectively) was higher than in the skin tissue (3734.73 and 5968.66 mg/kg FW for *M*. *sieversii* and ‘Tyroler Spitzlederer’, respectively). This was still the case at harvest for *M*. *sieversii* and by then also for *M*. *baccata*: 2510.39 and 1875.92 mg/kg for *M*. *sieversii* and *M*. *baccata* pulp, respectively, and 1430.10 and 1824.43 mg/kg FW in the skin, respectively. The compounds accumulated in the pulp and skin at the fruit development stage were predominantly chlorogenic acid (average of 1362.59 mg/kg FW) and procyanidin B2+B4 (average of 1032.56 mg/kg FW), respectively, for almost all the apple accessions considered in this study. The major phenolic compounds in the pulp of *M*. *sieversii* sampled during the fruit development stage was in fact procyanidin B2+B4 (1799.38 mg/Kg FW), while in the skin of the apple cultivar ‘Tyroler Spitzlederer’ the most abundant phenolic compound was the dyhydrochalcone phloridzin (1237.21 mg/kg FW). In contrast, at harvest the skin of all apple accessions had accumulated procyanidin B2+B4 (403.55 mg/kg FW), while for the pulp a bimodal distribution was observed. *M*. *sieversii*, the two clones of ‘Golden Delicious’ and ‘Braeburn’ had accumulated procyanidin B2+B4 (1133.79, 128.97, 159.48, 124.65 mg/kg FW, respectively), while the other three accessions (*M*. *baccata*, ‘Tyroler Spitzlederer’ and ‘Cripps Pink’) were characterized by a higher accumulation of chlorogenic acid (856.83, 216.76, 184.93 mg/kg FW, respectively). The dominant abundance of chlorogenic acid and procyanidin B2+B4 in apple was in agreement with the findings of Farneti et al.[49], who found a clear difference in phenolic compounds across the *Malus* genus, with the wild species generally having higher levels than the domesticated varieties. This difference was re-confirmed in this study when comparing the amount of phenolic compounds in both skin and pulp in fruit of the seven accessions sampled at harvest. However, at the fruit development stage, ‘Golden Delicious cl. Rugiada’ and ‘Tyroler Spitzlederer’ showed the highest concentration of phenolic compounds for the skin and pulp tissues, respectively (Fig 2 and S2 Table). These two varieties share a particular aesthetic property in their russeted skin, which is a genetically controlled disorder resulting from the periderm layer largely consisting of a network of suberized cells directly above the skin, appearing as a brown and rough matrix deposition[62,63]. This aliphatic polymer confers a rigid and corky aspect, which facilitates important water loss[64–67] due its permeability. A deficient outer cuticle and epicuticular wax composition might cause a relevant water loss with the consequent concentration of solutes, including phenolic compounds, as already proposed for apple by Gutierrez et al.[68].

**Fig 3:**
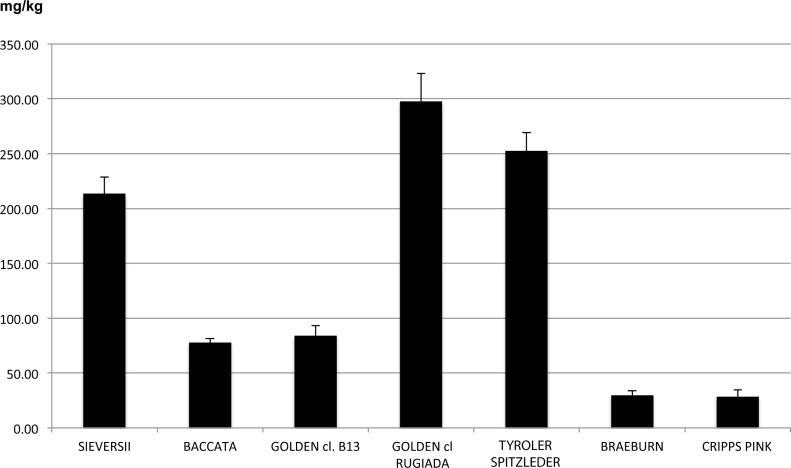
Phloridzin levels (mg/kg of fresh weight) in the skin of the seven apple accessions at harvest.

Focusing on individual compounds, the two russeted varieties, like the two wild species, were richer in procyanidin B2+B4 and phloridzin in the pulp at harvest, (S2 Table). A similar result was observed for chlorogenic acid in the skin tissue at harvest. However, the amount of phloridzin was higher for the two russeted accessions (‘Tyroler Spitzlederer’ and ‘Golden Delicious cl. Rugiada’) (297.75 and 252.68 mg/kg FW, respectively) than in the two wild species (213.49 and 77.84 mg/kg FW for *M*. *sieversii* and *M*. *baccata*, respectively). The effect of russeting on phenolic compound concentrations, such as phloridzin (Fig 3) observed here, is consistent with the findings of Gutierrez et al.[68]. This pattern can be appreciated more in detail in the direct comparison between the two clones of ‘Golden Delicious’ (‘cl. B13’ and ‘cl. Rugiada’). Although ‘Tyroler Spitzlederer’ is a russeted variety with a phenolic accumulation pattern similar to that of ‘Golden Delicious cl. Rugiada’, it has a different genetic background, making it difficult to clearly determine if the high concentration in secondary metabolites is due to a genetic factor or to the actual contribution of the russeted phenotype. The two ‘Golden Delicious’ clones, ‘cl. B13’ and ‘cl. Rugiada’, shared instead the same genetic background, hence the metabolic differences observed between the two samples can be only attributed to structural differences between the two skin types (S1 Fig). The higher concentrations of phenolic compounds in russeted skin agrees with observations by other authors, e.g. the higher concentration of polyphenols identified in ‘Renette Canada’[28]. Although this variety shows a russeting phenotype less intense compared with the two accessions of our study, ‘Renette Canada’ is actually the only russeted cultivar commercialized in the market, at least in Italy. This suggests that although russeting is generally considered a negative trait, it might also be of potential interest in future breeding programs aiming to ameliorate the concentration of phenolic compounds in apple fruit.

### Candidate gene based expression profiling

**Fig 4:**
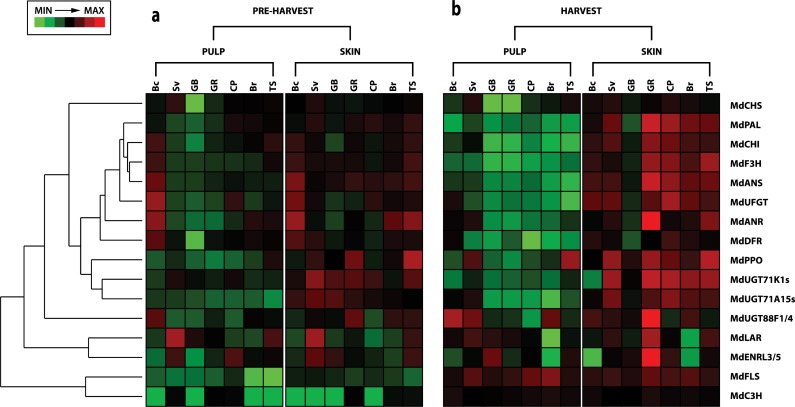
Gene expression profile. Hierarchical heat-map showing the gene expression of each gene for two tissues, pulp and skin, assessed during fruit development (a) and at harvest (b). BC (*Malus baccata*), SV (*Malus sieversii*), GB (‘Golden Delicious clone B’), GR (‘Golden Delicious clone Rugiada’), CP (‘Cripps Pink’), BR (‘Braeburn’) and TS (‘Tyroler Spitzlederer’). On the right side of the figure is indicated the code of the 16 genes employed in this study. The expression is reported through a color pattern, with green and red for low and high level of gene expression, respectively.

**Fig 5:**
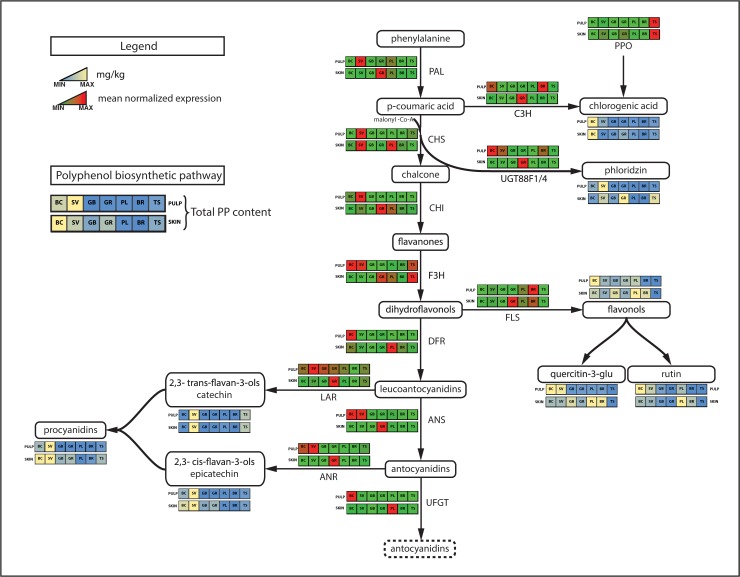
Scheme of the phenolic biochemical pathway. On the polyphenolic pathway depicted in the figure, the expression of the main genes at harvest together with the total accumulation of the major phenolic compounds are reported.

The collection of samples employed in this study to quantify the amount of phenolic compounds was further used to assess the expression of 16 genes involved in key steps of the phenolic biosynthetic pathway. *Phenylalanine ammonia lyase* (*MdPAL*), *chalcone synthase* (*MdCHS*), *chalcone-flavone isomerase* (*MdCHI*), *flavonoid 3-hydroxylase* (*MdF3H*), *dihydroflavonol 4-reductase* (*MdDFR*), *anthocyanin synthase* (*MdANS*) and *anthocyanidin 3-O-glucosyltransferase* (*MdUFGT*) were selected to represent the main pathway forming anthocyanins starting from phenylalanine. In addition, *leucoanthocyanidin reductase* (*MdLAR*) and *anthocyanidin reductase* (*MdANR*) were investigated to detect the expression of genes involved in the synthesis of procyanidins, while *flavonol synthase* (*MdFLS*), *UDP-dependent glycosyltransferase* (*MdUGT*) and *enoyl reductase* (*MdENLR*) were included as the main genes involved in the biosynthesis of flavonols and phloridzin, respectively. To better investigate the biosynthetic pathway of phloridzin, not yet completely elucidated, the expression of three different *MdUGT* genes (*MdUGT88F1/4*, *MdMdUGT71K1s* and *MdUGT71A15*), retrieved from Zhou et al.[69], was also assessed. In addition, the transcript accumulation of *MdENLR3/5* was considered for its role in the phloridzin production. According to Dare et al.[70], *ENLR* catalyzes the synthesis of phloridzin utilizing crotonyl-CoA and 2-decenoyl-ACP as substrate, which are structurally similar to the ploridzin precursor p-coumaroyl-CoA. From the biochemical pathway of chlorogenic acid, the expression of two elements was investigated, *p-coumaroyl ester 3-hydroxylase* (*MdC3H*), important for its accumulation, and the *polyphenol oxidase* (*MdPPO*), for its oxidation. This gene, in fact, has already shown an important role in encoding an enzyme reacting with chlorogenic acid during the development of the superficial scald disorder in apple[54,71]. The overall transcriptome analysis (Fig 4) showed that the genes assessed in this survey were more expressed in the skin tissue than in the pulp. This pattern was even more magnified at harvest stage. At the level of single gene expression, an important difference was observed between the two stages (S2 and S3 Figs). In the fruit development stage, 6 genes (*MdCHI*, *MdF3H*, *MdDFR*, *MdANS*, *MdUFGT* and *MdANR*) showed the highest expression in the wild species *M*. *baccata*, with no relevant difference between the two tissues (skin and pulp), except for *MdUFGT*, whose expression was considerably higher in the pulp (S2 Fig). In contrast, the other wild accession, *M*. *sieversii*, showed a higher gene expression for *MdCHS*, *MdFLS*, *MdLAR* and *MdENLR3/5*. In particular, while *MdCHS* was more expressed in the pulp tissue, *MdFLS* showed a higher transcript accumulation in the skin tissue. Beside the expression of these genes in the two wild accessions, it is also worth noting the expression profile detected in the group of the domesticated accessions. In particular, *MdPAL* and *MdF3H* showed the highest expression (across the entire comparison) in the skin of the russeted variety ‘Tyroler Spitzlederer’ (S2 Fig), which also showed the highest expression profile for *MdCHI*. In addition to this, ‘Golden Delicious cl. Rugiada’ showed the greatest expression for the gene *MdUGT88F1/4* in the skin tissue. This result further supported the hypothesis about the role of russeting in the final accumulation of phenolic compounds. This increase can also be attributed to an induced expression of genes involved in the secondary metabolites related pathway in skin-russeted accessions. This hypothesis finds support with the different expression pattern observed for *MdPAL*, *MdCHI*, *MdANR*, *MdPPO*, *MdC3H* and *MdUGT88F1/4* genes between the two clones of ‘Golden Delicious’. The effect of russeted skin is particularly evident in the expression profile of the *polyphenol oxidase gene* (*MdPPO*), which was expressed basically only in the skin of ‘Tyroler Spitzlederer’ and, at a lower level, ‘Golden Delcious cl. Rugiada’. The disrupted structure and biochemical diverse composition of the cuticle in russeted accessions might have therefore induced an oxidation process, consequently stimulating the expression of the *MdPPO* gene. At harvest, an even more dominant gene expression in the skin tissue was observed for almost the entire gene set, except for *MdFLS* and *MdC3H*, for which the highest transcription was detected in the pulp of ‘Braeburn’. The analysis of the gene expression also revealed that most of the elements showed the greatest expression in the skin tissue of ‘Golden Delicious cl. Rugiada’, except for *MdUFGT* and *MdUGT71A15s*, for which the highest expression was detected in the skin of ‘Cripps Pink’ (S2 and S3 Figs). The comparison of the overall expression profile of the 16 genes between the two sampling stages (fruit development and harvest) revealed two major differences. At harvest, the highest expression in the wild accessions was only for the *MdCHS* gene in *M*. *sieversii* (S3 Fig). For the other genes, the two wild accessions showed a consistent low expression level, similar, if not lower, to the domesticated varieties. The second aspect important to underline is about the highest expression detected at harvest for 9 out of 16 genes (*MdPAL*, *MdCHI*, *MdF3H*, *MdANR*, *MdFLS*, *MdENLR3/5*, *MdANS*, *MdC3H* and *MdUGT88F1/4*), with a fold change ranging from 3.8 (for *MdUGTF88F1/a*) to 58 (for *MdFLS*). The discrepancy between the expression of genes involved in phenolic compound biosynthesis and the accumulation of related secondary metabolites should depend by other aspects, such as skin russeting or fruit size[68]. The higher concentration of phenolics in the two wild accessions agrees with their increased gene expression through the polyphenolic pathway (Fig 5), which showed a higher expressions for the *MdF3H*, *MdLAR*, *MdC3H* and *MdPPO* genes in the pulp of the two wild accessions together with ‘Braeburn’ and ‘Tyroler Spitzlederer’. In this figure is moreover underlined the contribution of the russeting phenotype in regulating the expression of these genes. For simplicity, only the expression profile of *MdUGT88F1/4* is shown as a representative element of the phloridzin biosynthetic pathway (Fig 5), because of its well-known role in the production of this dihydrochalcone[69]. All the three UGT genes analyzed in this work belong to phylogenetic group 5[69], distinguished by their phloretin 2’-O-glycosiyltransferase activity. Moreover, *UGT71K1s* and *UGT71A15* also seem to be able to catalyze the conversion of phloretin into trilobatin.

### The role of fruit size in the final accumulation of phenolic compounds in apple

**Fig 6:**
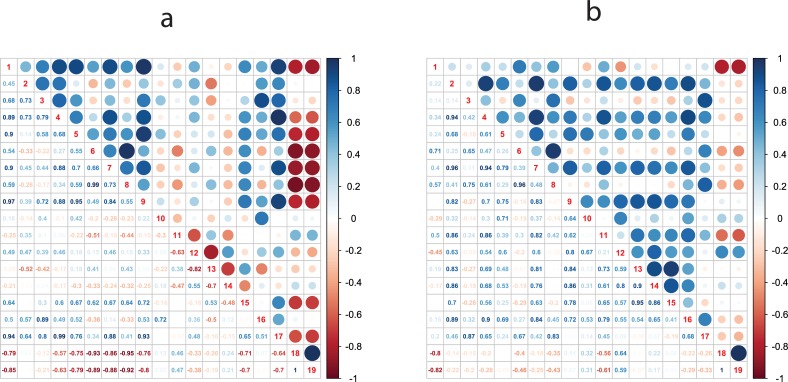
Correlation plot analysis. The correlation between the expression pattern of the 16 genes together with the total phenolic content and fruit size (defined by diameter and weight) is presented for both pulp (a) and skin (b) tissues, respectively. The correlation value is indicated by color (blue for positive correlation and red for negative correlation), while the magnitude of correlation is indicated two-fold, with the size and color intensity of the dots in the upper-right part of the plot and with numbers in the lower-left part of the plot. For each plot, the numerical code in the diagonal stands for 1: total phenols, 2: *PAL*, 3: *CHS*, 4: *CHI*, 5: *F3H*, 6: *DFR*, 7: *ANS*, 8: *UFGT*, 9: *ANR*, 10: *PPO*, 11: *FLS*, 12: *LAR*, 13: *C3H*, 14: *ENRL-3-5*, 15: *UGT88F1/4*, 16: *UGT71K1s*, 17: *UGT71A1s1*, 18: fruit diameter, 19: fruit weight.

**Fig 7:**
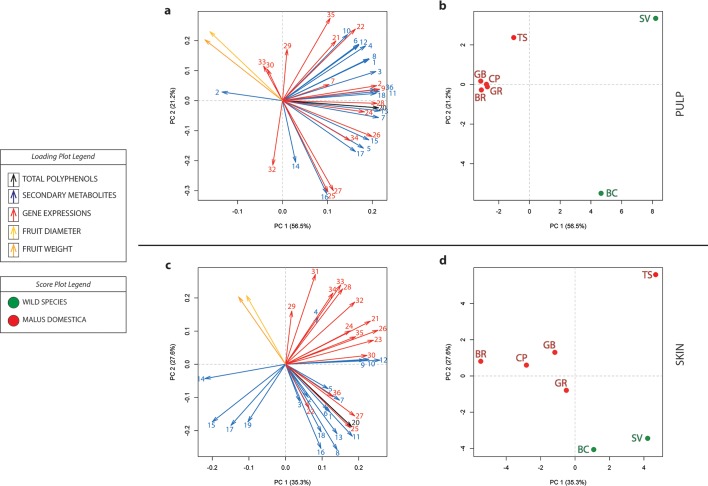
2D-PCA plot of the metabolite and gene expression pattern of the seven apple accessions assessed at harvest. The loading plots related to fruit size, expression of genes and secondary metabolites for the pulp and skin are illustrated in panel a and c, respectively. The score plots for these two tissues (b and d, respectively) depict instead the distribution of the samples, distinguished by color (green for wild species and red for *M*. x *domestica* accessions) and code, BC: *Malus baccata*, SV: *Malus sieversii*, GB: ‘Golden Delicious clone B’, GR: ‘Golden Delicious clone Rugiada’, CP: ‘Cripps Pink’, BR: ‘Braeburn’, TS: ‘Tyroler Spitzlederer’. For panel a and c the identity of the numerical code for each vector is reported in the S4 Table.

The role of fruit size in the accumulation of secondary metabolites was highlighted in the correlation plot illustrated in Fig 6, which clearly showed that fruit weight and diameter are negatively correlated with the total phenolic concentration (-0.79 and -0.85, respectively, in the pulp, and -0.80 and -0.82 in the skin). The contribution of the fruit size in the general phenolic concentration was also depicted in the PCA analysis carried out for the two tissues collected at harvest, the pulp (Fig 7A and 7B) and the skin (Fig 7C and 7D). For both types of tissues, fruit diameter and weight were projected with an opposite or orthogonal orientation with regards to the gene expression and the metabolite accumulation assay, respectively (Fig 7A and 7C), but with a slightly different distribution of the accessions over the 2D-PCA plot (Fig 7B and 7D). In pulp tissue, the PC1 (contributing to 56.5% of the total variance) clearly distinguished the two wild accessions from the group of the domesticated varieties (Fig 7B). This difference was much lower in the skin tissue (Fig 7D), with the PC1 explaining a smaller portion of the total variance (35.3%). In this plot it is however interestingly to note that ‘Golden Delicious cl. Rugiada’ was more closely plotted to *M*. *baccata* and *M*. *sieversii*, while ‘Tyroler Spitzlederer’ was plotted with a similar PC1 value as *M*. *sieversii*, but in the opposite, positive end of the PC2 quadrant. The total polyphenolic concentration of both wild and domesticated accessions can be, therefore, influenced by both fruit size and skin russeting. While the increased fruit size of the fruit of the *M*. *domestica* accessions can have contributed to a decrease in the polyphenolic concentration due to a dilution effect driven by the fruit cell enlargement, the increased concentration observed in russeted-accessions could have been determined by a dehydration process together with a gene activation triggered by this particular phenotype.

### Polyphenolic comparison between white-fleshed and red-fleshed apples

**Fig 8:**
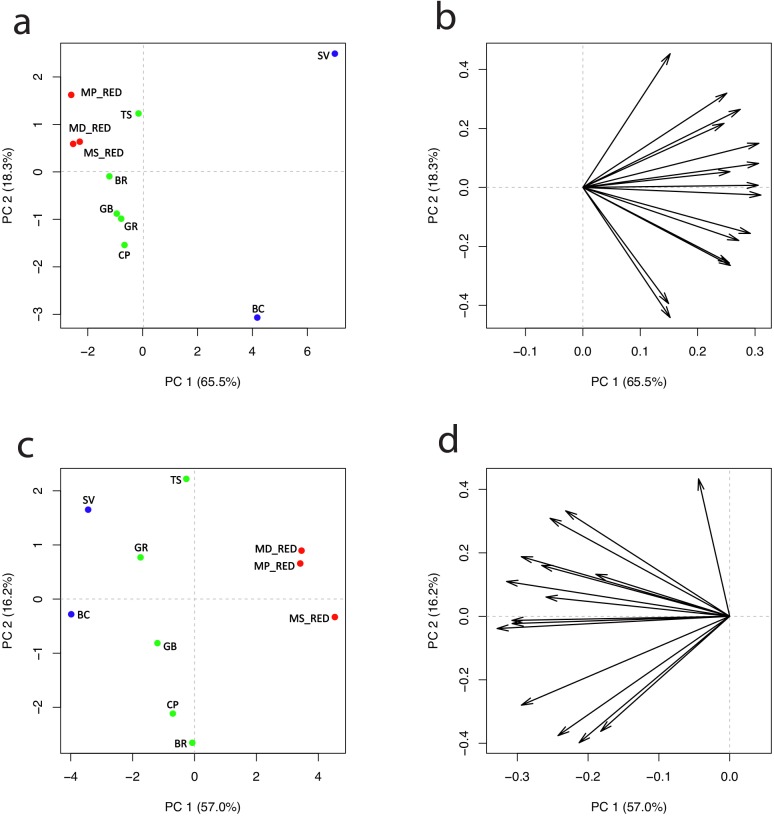
2D-PCA plot of the secondary metabolite accumulation in the white-fleshed and red-fleshed groups of apples. The distribution of the three red-fleshed accessions compared with the seven white-fleshed accessions for secondary metabolite accumulation is illustrated for the pulp (a) and the skin (c), respectively. Each sample is distributed according to the orientation of each compound illustrated in the two loading plots, for both pulp (b) and skin (d). Each sample is identified with a color (blue: white-fleshed wild apple accessions, green: white-fleshed domesticated accessions, red: red-fleshed accessions) and code (MD_RED: *Malus* x *domestica* red-fleshed, MP_RED: *Malus pumila* red-fleshed, MS_RED: *Malus sylvestris* red-fleshed, SV: *Malus sieversii*, BC: *Malus baccata*, TS: ‘Tyroler Spitzlederer’, GR: ‘Golden Delicious cl. Rugiada’, GB: ‘Golden Delicious cl. B’, BR: ‘Braeburn’, CP: ‘Cripps Pink’).

One of the most advanced efforts to increase the bio-availability of phenolic compounds in apple is the breeding for red-fleshed apple. Red flesh is a feature naturally present within the apple germplasm, especially in wild species. A *MYB* transcription factor (*MYB10*) was identified as the main key element responsible for the increased accumulation of anthocyanin in several organs of the plant, including fruit, throughout the activation of the anthocyanin pathway genes[70–74]. Because of the distinct difference in anthocyanin concentration between red- and white-fleshed apples (especially in cyanidins) and the availability of molecular markers suitable for the assisted selection of this trait, red-fleshed apples became one of the most interesting possibilities for improving this fruit for nutraceutical value[50]. The metabolite analysis presented by Espley et al., although providing important results, is however focused on the comparison between ‘Royal Gala’ (white-fleshed and red-skin apple) and *MYB10* transgenic lines. In this work we investigated the phenolic content of seven apple accessions considering a wide array of molecules than solely anthocyanins. This class of compounds is basically negligible in the apple cultivars to date available on the market, since they all belong to the white type of flesh. To this end, the concentration of the phenolic compounds listed till now has been compared with three red-fleshed apple accessions. Like the set of white-fleshed apples, the red-fleshed apples varied in fruit size according to the species they belonged to. The *M*. *domestica* and *M*. *pumila* fruit showed an average weight of 80.8 and 75.1 g, and a fruit diameter of 6 and 5.8 cm, respectively. *M*. *sylvestris*, in contrast, showed an average weight of 17.7 g and were about half the size (3.1 cm) compared with the other two accessions. The analysis of all phenols (S3 Table), except anthocyanins, identified 15 phenolic compounds commonly shared between the two flesh colour groups of apples, such as neochlorogenic acid, chlorogenic acid, *trans*-piceide, *cis*-piceide, catechin, epicatechin, procyanidin B1, procyanidin B2+B4, quercetin-3-Rha, kampferol-3-rutinoside, quercetin-3-galactoside+glucoside, isorhamnetin-3-glucoside, rutin, arbutin and phloridzin. The effect of fruit size on the final concentrations of the polyphenols was validated for certain compounds in the red-fleshed apples. Especially for chlorogenic acid, epicatechin and procyanidin B2+B4, red-fleshed *M*. *sylvestris* showed a higher concentration (90.2, 133 and 16.9 mg/kg FW, respectively) with a fold-change ranging from 1.3 to 4.5 with regards to the other two red-fleshed accessions (S3 Table). Comparing the metabolite profiles of the two groups, it is interesting to note that the higher accumulation in phenolic compounds was observed in the white-fleshed apples. The fold-change was similar for both flesh and skin tissues (S4A and S4B Fig), while at harvest, the higher fold-change was apparent in the comparison with the wild accessions rather than the group of the domesticated ones. The different accumulation levels in polyphenolic compounds in the red- and white-fleshed apples were clearly visualized in the PCA plots computed for flesh (Fig 8A and 8B) and skin (Fig 8C and 8D), respectively. PC1, which explained the overall phenolic accumulation, accounted for 65.5% and 57% of the entire variability for flesh and skin (Fig 8A and 8C), respectively, and distinguished the wild white-fleshed species from the domesticated accessions and the group of red-fleshed accessions. This result reflects the fact that the red-fleshed apples were obviously richer in anthocyanins than the white-fleshed apples, but also that the white-fleshed apples were richer in other polyphenols. The comparison of polyphenolic accumulation in the pulp of the white-fleshed domesticated and red-fleshed accessions revealed a fold-change of 17.5, 12.7 and 26.2, respectively, for procyanidins B1 and B2+B4, and quercetin-3-Rha. This specific accumulation pattern can be explained by the fact that the red coloration is induced by the activation of a *MYB* transcription factor[72], which activates the anthocyanin-related genes in red-fleshed apples. It is worth noting that the low accumulation of phenolic compounds, except for anthocyanins in red-fleshed apples, was observed both in pulp (where the difference is obvious) and skin. This result can have an important impact on the amelioration of the nutraceutical properties of apple. In fact, although it is widely documented that anthocyanins play important antioxidant roles, for example inhibiting the growth of cancerous cells as well as inflammation processes, their bioavailability is much lower than that of other flavonoids[75,76]. In human studies it has been discovered that anthocyanins are in fact quickly degraded or rapidly absorbed and excreted[77]. Beside anthocyanins, apple fruit, as highlighted in this study, are particularly rich in procyanidins, which are a category of proanthocyanidins, exclusively consisting of (epi)catechins, especially B-type procyanidins formed by (+)- and (-)-catechin. These classes of metabolites play a crucial role in protecting the human health by preventing chronic diseases due to their antioxidant role[78]. Their bioavailability is determined by their degree of polymerization, with mono-trimers being rapidly absorbed. Procyanidins, which pass unaltered through the stomach, are then degraded by the gut microbiota (especially in the colon), thus exerting a prebiotic-like effect[79]. It is therefore important to have a more informative and detailed cataloguing of the array of beneficial components present within fruit, and linking this information with clinical surveys about their metabolism in the human system.

## Conclusions

The interest of the scientific community for plants secondary metabolites has increased considerably in the last decade because of the evidence of their beneficial impact on the human health. In this research, the accumulation of these nutraceutical compounds in two tissues of apple fruit (skin and pulp) was assessed both during the fruit development and at harvest. While confirming that the concentration of these phytochemicals is higher in wild species, we demonstrated that fruit characteristics such as skin russeting and fruit size might strongly affect the phenolic accumulation in the fruit. The breeding programs historically focused on aesthetic (skin color and fruit size) and postharvest related traits (such as fruit texture), should now consider the increasing level of nutraceutical compounds, in order to release ideotypes distinguished by a superior fruit quality and health promoting properties. If small-fruited wild accessions are selected as donor parents for improving the levels of these compounds, one needs to consider that subsequent selection steps to increase fruit size (the most important aesthetic features for commercial purposes) can dilute the concentration of phenolic compounds. Alternatively, the concentration of phenolic compounds can be improved by selecting apples with russeted skin. Although russeting is generally considered as a negative trait, particular varieties, such as ‘Renette Canada’, are appreciated for these properties. This accession is, however, triploid, therefore less suitable in breeding programs than russeted diploid varieties, such as ‘Tyroler Spitzlederer’ or ‘Golden Delicious cl. Rugiada’, provided the trait is heritable.

## Supporting information

S1 Fig Skin feature of three apple cultivars: smooth skinned ‘Golden Delicious clone B’ (a), and two russet-skinned accessions: ‘Golden Delicious clone Rugiada’ (b) and ‘Tyroler Spitzlederer’ (c).
(JPG)

S2 Fig Expression pattern of phenolic related genes at fruit development stage. For each gene, the expression profile is illustrated as Mean Normalized Expression on the y-axes, while on the x-axes the samples assessed are reported. Each accession is indicated by a different color and code as follow: BC (dark grey) *Malus baccata*, SV (light grey) *Malus sieversii*, GB (blue) ‘Golden Delicious clone B’, GR (orange) ‘Golden Delicious clone Rugiada’, CP (red) ‘Cripps Pink’, BR (green) ‘Braeburn’, TS (yellow) ‘Tyroler Spitzlederer’. The two tissues are indicated as P (solid bar) for pulp and S (wide upward diagonal bars) for skin.
(PDF)

S3 Fig Expression pattern of phenolic related genes at harvest stage. For each gene, the expression profile is illustrated as Mean Normalized Expression on the y-axis, while on the x-axis the samples assessed are presented. Each accession is indicated by a different color and code as follow: BC (dark grey) *Malus baccata*, SV (light grey) *Malus sieversii*, GB (blue) ‘Golden Delicious clone B’, GR (orange) ‘Golden Delicious clone Rugiada’, CP (red) ‘Cripps Pink’, BR (green) ‘Braeburn’, TS (yellow) ‘Tyroler Spitzlederer’. The two tissues are instead indicated as P (solid bar) for pulp and S (wide upward diagonal bars) for skin.
(PDF)

S4 Fig Fold change of the difference in phenolic content between the white-fleshed and the red-fleshed groups of apples assessed in the pulp (panel a) and skin (panel b) tissues, respectively. With blue histograms are indicated the fold-change for each of the 15 phenolic compounds commonly shared between the white-fleshed wild accessions and the red-fleshed accessions. With red histograms, instead, are depicted the fold-change between the white-fleshed domesticated varieties and the red-flesh apples.
(PDF)

S1 Table List of primers used in the RT-qPCR analysis. For each element, the acronym (gene), gene identity (identity), the gene ID (MDP), the oligo name and the relative sequences are reported.
(PDF)

S2 Table Phenolic compound assessment in apple. For each of the 19 metabolites is reported the amount (in mg/kg of fresh weight) in both pulp and skin tissues collected from the fruit of the seven white-fleshed apple accessions at the two sampling stages, fruit development and harvest. n.d.: not determined.
(XLSX)

S3 Table Phenolic compound quantification in pulp and skin of three red-fleshed apple accessions. For each of the 15 metabolites in common with the group of the white-fleshed apples, the amount of phenolic compounds is reported as mg/kg. n.d.: not determined.
(XLSX)

S4 Table Identity for each loading vector illustrated in Fig 7. (XLSX)
