# Supplementary figures and images for: Multifaceted analyses disclose the role of fruit size and skin-russeting in the accumulation pattern of phenolic compounds in apple
Source: PLoS One. 2019 Jul 15;14(7):e0219354. doi: 10.1371/journal.pone.0219354 (PMC6629076; doi:10.1371/journal.pone.0219354)

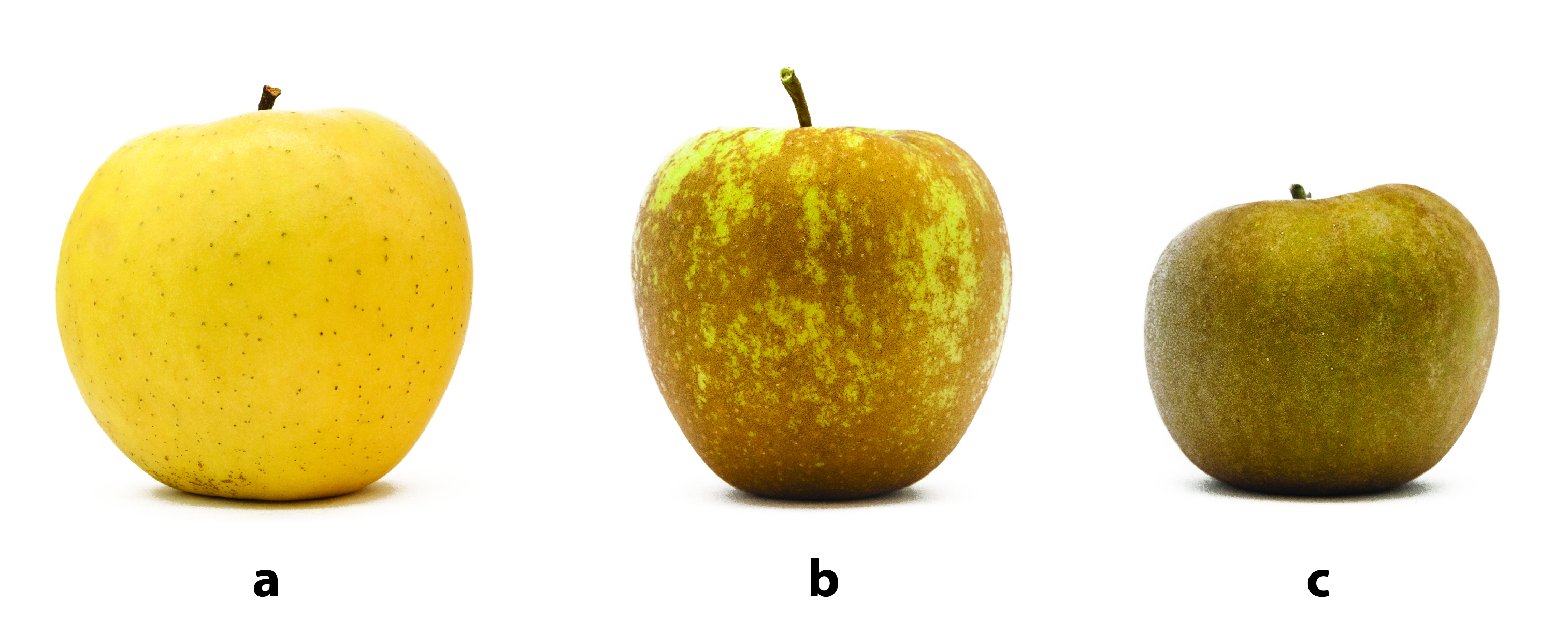

Supplement: S1 Fig — Skin feature of three apple cultivars: smooth skinned ‘Golden Delicious clone B’ (a), and two russet-skinned accessions: ‘Golden Delicious clone Rugiada’ (b) and ‘Tyroler Spitzlederer’ (c). (JPG) [file pone.0219354.s001.jpg]

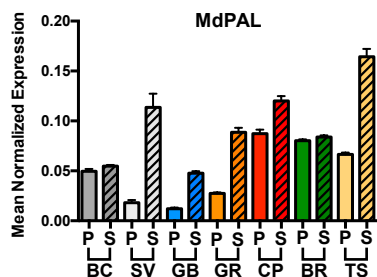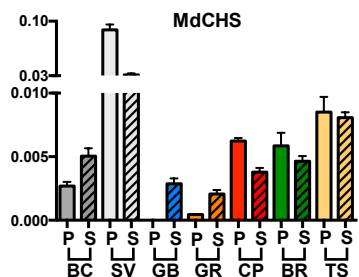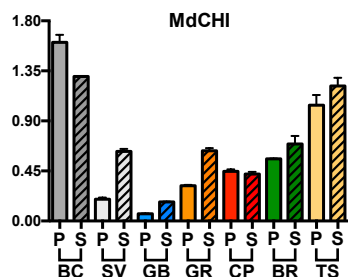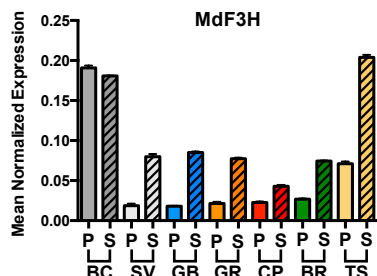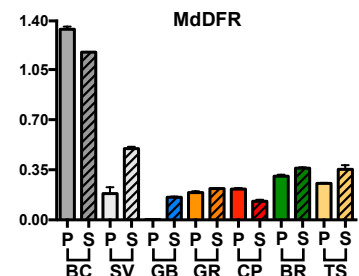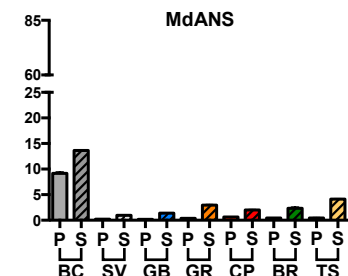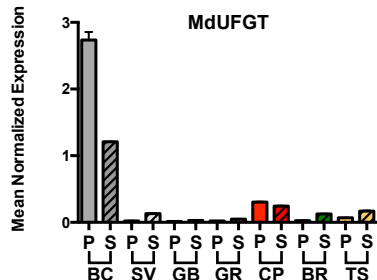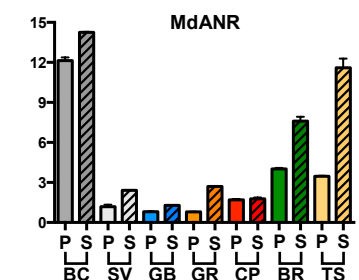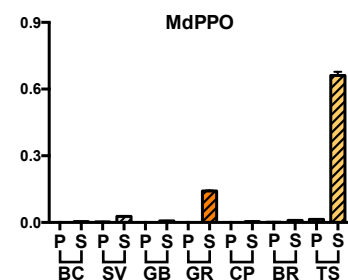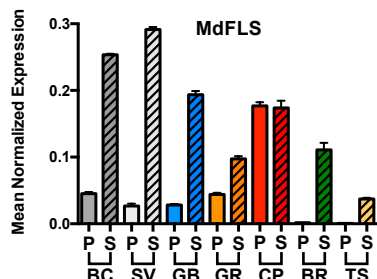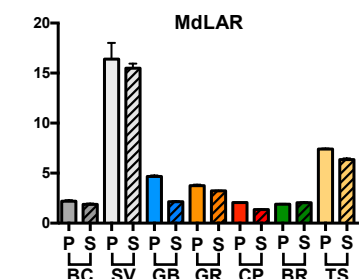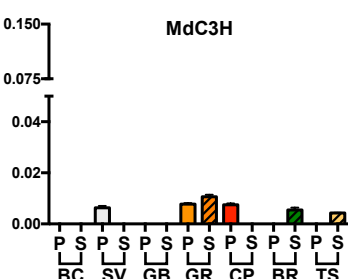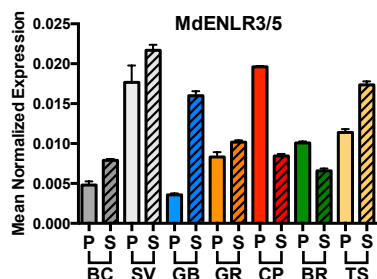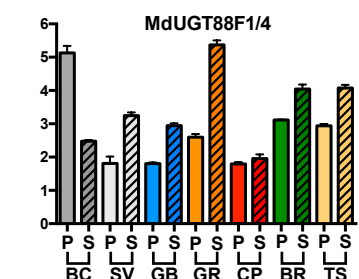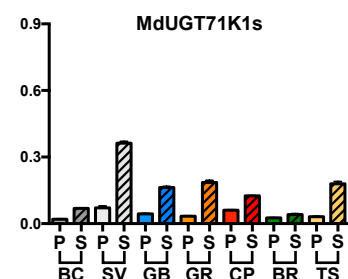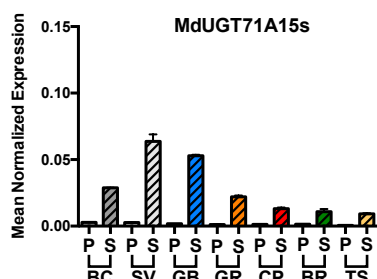

Supplement: S2 Fig — For each gene, the expression profile is illustrated as Mean Normalized Expression on the y-axes, while on the x-axes the samples assessed are reported. Each accession is indicated by a different color and code as follow: BC (dark grey) Malus baccata, SV (light grey) Malus sieversii, GB (blue) ‘Golden Delicious clone B’, GR (orange) ‘Golden Delicious clone Rugiada’, CP (red) ‘Cripps Pink’, BR (green) ‘Braeburn’, TS (yellow) ‘Tyroler Spitzlederer’. The two tissues are indicated as P (solid bar) for pulp and S (wide upward diagonal bars) for skin. (PDF) [file pone.0219354.s002.pdf]

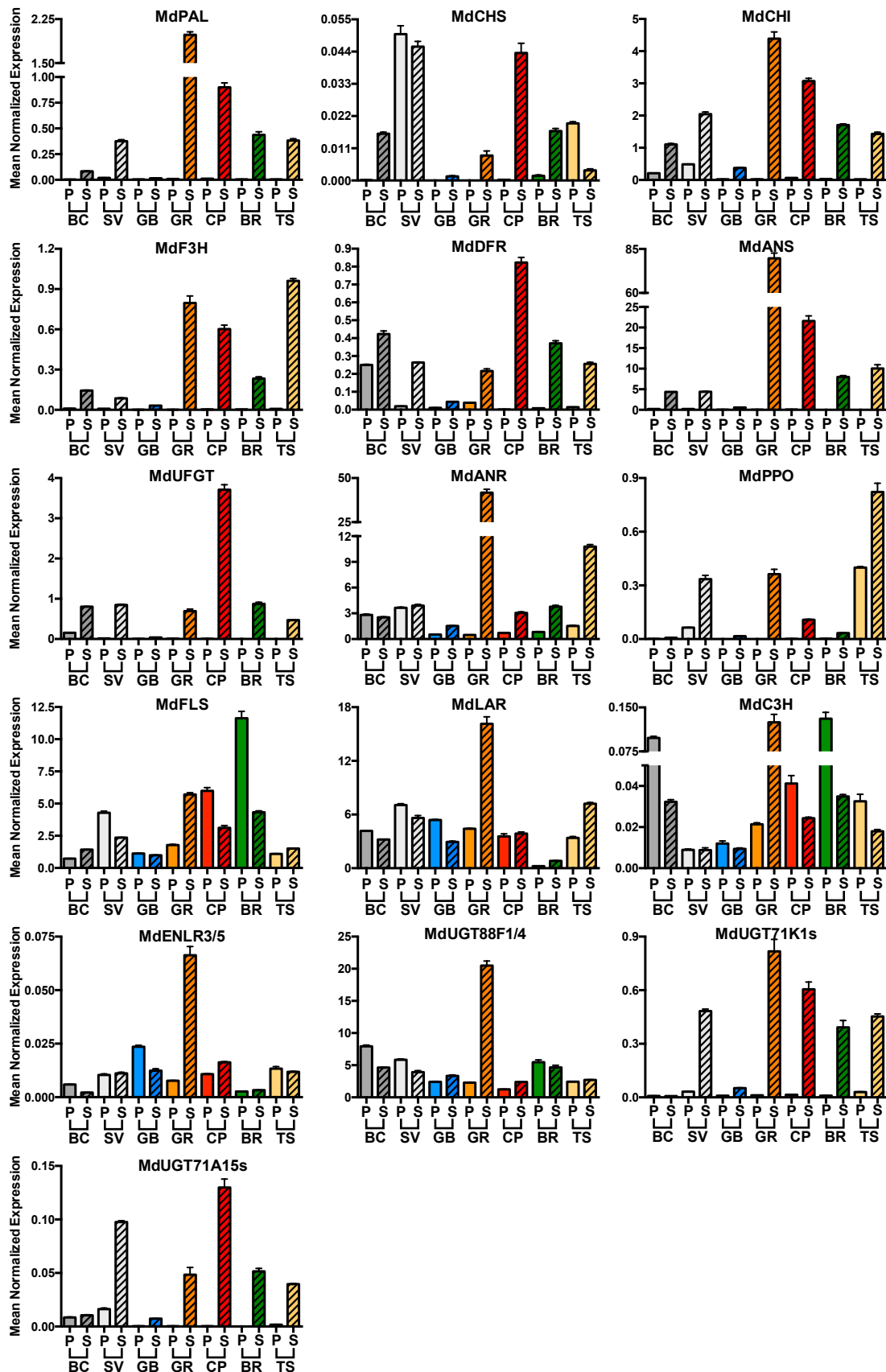

Supplement: S3 Fig — For each gene, the expression profile is illustrated as Mean Normalized Expression on the y-axis, while on the x-axis the samples assessed are presented. Each accession is indicated by a different color and code as follow: BC (dark grey) Malus baccata, SV (light grey) Malus sieversii, GB (blue) ‘Golden Delicious clone B’, GR (orange) ‘Golden Delicious clone Rugiada’, CP (red) ‘Cripps Pink’, BR (green) ‘Braeburn’, TS (yellow) ‘Tyroler Spitzlederer’. The two tissues are instead indicated as P (solid bar) for pulp and S (wide upward diagonal bars) for skin. (PDF) [file pone.0219354.s003.pdf]

a

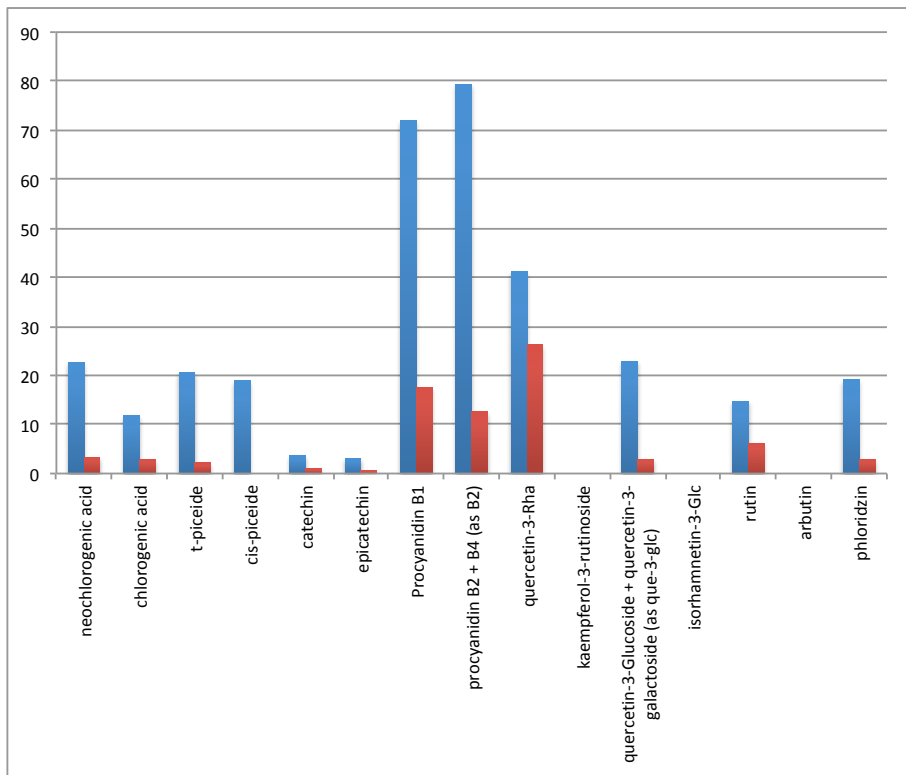

b

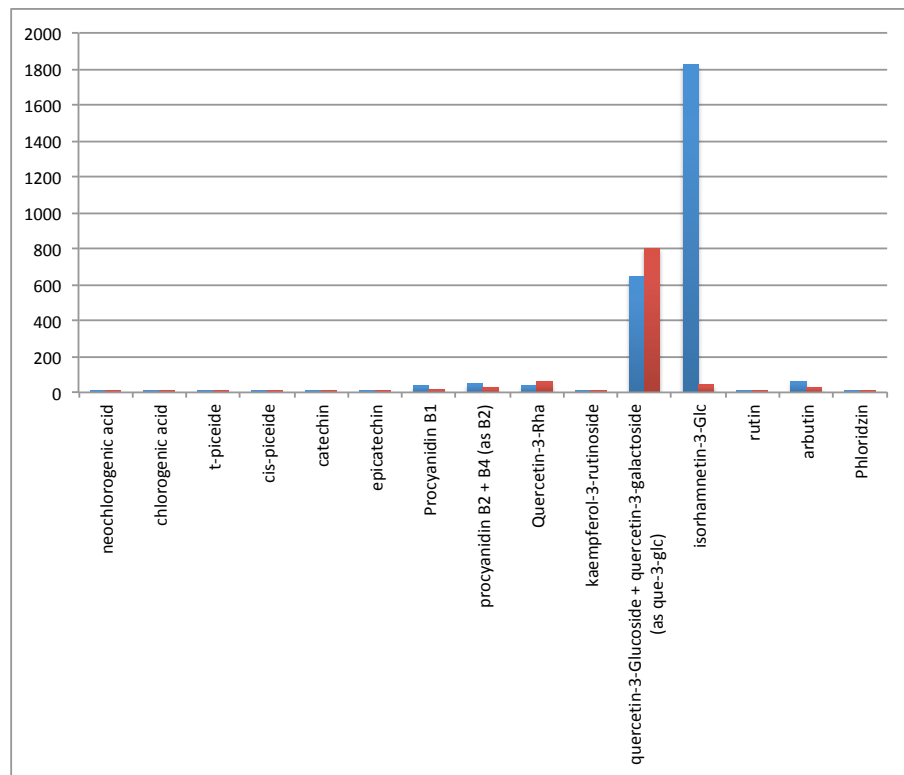

Supplement: S4 Fig — Fold change of the difference in phenolic content between the white-fleshed and the red-fleshed groups of apples assessed in the pulp (panel a) and skin (panel b) tissues, respectively. With blue histograms are indicated the fold-change for each of the 15 phenolic compounds commonly shared between the white-fleshed wild accessions and the red-fleshed accessions. With red histograms, instead, are depicted the fold-change between the white-fleshed domesticated varieties and the red-flesh apples. (PDF) [file pone.0219354.s004.pdf]
