# Supplementary material for: Multifaceted analyses disclose the role of fruit size and skin-russeting in the accumulation pattern of phenolic compounds in apple
Source: PLoS One. 2019 Jul 15;14(7):e0219354. doi: 10.1371/journal.pone.0219354 (PMC6629076; doi:10.1371/journal.pone.0219354)
Supplement: S1 Table — For each element, the acronym (gene), gene identity (identity), the gene ID (MDP), the oligo name and the relative sequences are reported. (PDF) [file pone.0219354.s005.pdf]

gene	identity	MDP	OLIGO NAME	SEQUENCE
MdPAL	phenylalanine ammonia lyase	MDP0000191304	MdPAL_for	AGACCCTCAATGCCTCAGAA
			MdPAL_rev	CAAGCCAGAACCAACAGCAG
MdCHS	chalcone synthase	MDP0000686666	MdCHS_for	CGCTTCTCTTAACATTGTGTCAC
			MdCHS_rev	GAACTTCCTCGACTGTAACCATT
MdCHI	chalcone isomerase	MDP0000759336	MdCHI_for	CCGGAAGCTACAAATGCGGTGATA
			MdCHI_rev	GGATAACCTCGCGGCCAAACTT
MdF3H	flavone 3-hydroxylase	MDP0000704377	MdF3H_for	TGGGGTTGGATACAGAGGCATTGA
			MdF3H_rev	TTAGGTCGGGCTGAGGGCATTTT
MdDFR	dihydroflavonol 4-reductase	MDP0000494976	MdDFR_for	AGGGCATCGACGACAACTTAGAA
			MdDFR_rev	CGGAATCGGAATCGGAATCA
MdANS	anthocyanidin synthase	MDP0000360447	MdANS_for	TTCTGTCACTTGGCTTGGGATTGG
			MdANS_rev	CGGCTGAGGGCATTTTGGGTAG
MdC3H	p-coumarate 3-hydroxylase	MDP0000466557	MdCH3_for	AGCGATGGGAGGGAAGGGTTGT
			MdCH3_rev	GTGGATCGGATATTTTAGGTGGTG
MdFLS	flavonol synthase	MDP0000260404	MdFLS_for	GAGTTAAGGCTGCTGGTGGTGATA
			MdFLS_rev	GTTGGGGACGAGAATGGTGACG
MdLAR	leucoanthocyandin reductase	MDP0000376284	MdLAR_for	CGATTGCTTCTTGGCCCTACTTTG
			MdLAR_rev	TTCGCATGTCGTCCACCGTTTTC
MdANR	anthocyanidin reductase	MDP0000243194	MdANR_for	GGGTACCCTGCCTCCAAGACACTA
			MdANR_rev	AGAGAAGGACCGGCCATAAGAGAA
MdPPO	polyphenol oxidase	MDP0000699845	MdPPO_for	CCTACTCACAAAGCCCAAGC
			MdPPO_rev	CCTCCAAGACCAAGAAGCAC
MdUGT88F1/4	phloretin 2'-O-glucosyltransferase	MDP0000219282	MdPTG_for	CCAAGTCGAAGCGTGCTG
			MdPTG_for	GCTATCTCCTTCAGTTGAGCA
MdUGT71K1s	UDP-glycosyltransferase 71K1	MDP0000163017	MdUGT71K1s_for	CATCACCAACCTCGTCTCCT
			MdUGT71K1s_rev	GCCACATCAATCATGGACACA
MdUGT71A15	UDP-glycosyltransferase 71A15	MDP0000215525	MdUGT71A15s_for	GCCCAACCGGACAAACAAGA
			MdUGT71A15s_rev	GCAAGCCGAGGCTTCGAC
MdENLR3/5	enoyl reductase	MDP0000207724	MdENRL3/5_for	AATGACAATTTTAAGGTGGCTTCA
			MdENRL3/5_rev	TGCCCTTGTAACAAACTTATCCA
MdUFGT	flavonoid 3-O-glycosyltransferase	MDP0000478252	MdUFGT_for	CCACCGCCCTTCCAAACACTCTC
			MdUFGT_rev	GCACCCCGTCAGCCACATC
Md8283			Md8283_for	CTCGTCGTCTTGTTCCCTGA
			Md8283_rev	GCCTAAGGACAGGTGGTCTATG
